# Sub-Glottic Papillomata

**Published:** 1888-12

**Authors:** Frank O. Stockton

**Affiliations:** Atlanta, Ga., Late Professor of Laryngology in College of Physicians and Surgeons and Chicago Polyclinic, Chicago, Illinois


### SUB-GLOTTIC PAPILLOMATA.*

BY FRANK O. STOCKTON, M. D., ATLANTA, GA.,

Late Professor of Laryngology in College of Physicians and Surgeons and Chicago Polyclinic, Chicago, Illinois.

No branch of medical science has made such great strides in the past quarter century as that of diseases of the nose and throat. Previous to the discovery of the laryngoscope little or nothing was known of the neoplasms of the larynx. All operations directed against any trouble in this organ required an opening into the larynx itself and they were more often fatal than otherwise; whereas, the brilliant results of intra-laryngeal surgery in the present day are certainly astonishing.

Of all the operations those directed against sub-glottic disease are the most difficult and give the most brilliant results. It is a fortunate circumstance that growths in this region are of rare occurrences, owing to the difficulty of their recognition and removal.

*Read before the Atlanta Society of Medicine, September 21, 1888.

The predisposing causes of sub-glottic papilloma is without doubt a chronic congestion, or, more properly speaking, a chronic catarrhal difficulty. Constitutional diseases, such as tuberculosis or syphilis, do not appear to favor true growths.

Oertel and Paul Bruns divide the papillomata into three varieties, which show a marked distinction in their characteristics. The first variety is a dark-red tumor, varying in size, with slightly irregular surface and a broad base. This form does not recur at all or only after a long interval. The second variety is a grayish white, very warty growth, starting usually from the vocal cords from a broad base; these also recur slowly. The third variety are large, reddish tumors resembling the mulberry and usually multiple. This variety is most frequent in children and is very liable to recurrence. The symptoms of these difficulties may be divided into functional and physical, the functional being found in the voice, dyspnœa pain and difficulty in swallowing; the physical in such signs as are furnished by the laryngoscope, probe, finger and other methods of examination. The changes in the voice may be slight or very great; frequently there is only a change of pitch, in other cases complete aphomia, so that except to the experienced ear it is of but little value. It is usually the case that difficulty of breathing precedes the loss of voice. The difficulty of breathing may be slight at times and then on the least exertion become very great. Pain is seldom present to any great extent and usually only when there is ulceration. Difficulty in swallowing is also a symptom that is frequently present. This symptom may be caused in two ways. The size and position of the tumor may be such as to press on the œsophagus, or the tumor may prevent the closure of the glottis so that fluids pass into the larynx. The laryngoscope enables us to tell the form, size, color and attachments of the tumor. By the probe and finger we determine its consistency. Of course experience is needed to correctly classify and value the information derived from the various signs and symptoms. The prognosis with regard to life is good, but of necessity better in adults than children.

In regard to the voice, if the growth can be removed through the natural passages, usually a favorable prognosis can be given, but the liability of recurrence must not be forgotten. The treatment is divided into palliative and radical. The only safe palliative treatment is either tracheotomy or intubation, and should only be resorted to when the patient's health is such as to preclude a radical operation, or when the growth cannot be removed through the natural passages and the patient objects to an extra-laryngeal operation. Radical treatment may be performed in two ways: either through the natural passages or by direct openings into the larynx. The endo-laryngeal operation is always preferable, as there is no danger, little or no pain, seldom any loss of blood and the results immediately perceptible. Operations by this method formerly required considerable special training to be given the patients previous to the operation so that they would become used to the passages of instruments, but by using cocaine the necessity for this training has been overcome. The operator must, of necessity, be thoroughly familiar with the parts and the instruments and also be possessed of considerable manual dexterity. It is easily understood that a large growth is more easily removed than a small one, owing to the great difficulty frequently encountered in grasping the small one. Then a growth situated in the posterior portions of the larynx is more easily grasped than one anteriorly placed. The extra-laryngeal method should never be used unless absolutely impossible to remove it by the other method, owing to the great danger to life either through primary or secondary hemorrhage, pneumonia, pleurisy or metastatic abscess of the lungs. Then the great danger of permanent injury to the voice must not be forgotten. Out of a number of cases treated during the past two years, two have been selected as being of special interest. The first was of the large red variety with smooth surface and broad base. The second was of the mulberry variety and multiple. The first occurred in a young lady of thirteen, of good health generally, but had suffered from repeated colds, producing a chronic catarrhal condition of the air passages. A year previous to the time I saw her, it was noticed that there was a change in her voice and that at times she would become quite hoarse. This condition of affairs continued to grow worse until at the time I saw her she spoke only in a hoarse whisper, and also suffered from slight difficulty in breathing, when she exerted herself at all, and had with this a slight cough. On examination the fauces and pharynx were found very much congested with numerous enlarged fallicles. The laryngoscope showed that the larynx, including the vocal chords, was congested, but not enough to cause any such symptoms. Below the glottis and anteriorly situated appeared a smooth, red body, shutting off considerable of this passage and extending upward until it interfered with the action of the cords. By means of the probe I found that the growth was attached by a broad base and to the cricoid cartilage. It was not tender to the touch nor did it bleed readily. It was exceedingly difficult to reach even with so light and delicate an instrument as the probe. I decided to attempt its removal the next morning, telling the patient to take nothing for breakfast except a glass of milk, and gave her some coca troches to take in order to allay the irritable condition of the fauces and pharynx. In the morning I applied a 20 per cent. solution of the hydrochlorate of cocaine, waiting ten minutes for it to take effect, then after repeated efforts succeeded in grasping it with a pair of McKenzie's forceps, but failed to bring it away after using all the force I deemed advisable. I then took a pair of strong laryngeal cutting forceps and succeeded in cutting most of it off. The hemorrhage was slight, as the forceps were dull and did not make a clean cut. I easily stopped the flow of blood by holding a piece of cotton against the part. Immediately on allowing the patient to use her voice it was found to be clear and strong. The patient was then allowed to go home for the day, telling her to call me at once should she become hoarse. I did this because I had some fear that there might be some œdema following such severe measures. Everything moved along without difficulty and the next day I removed the remainder of the growth and touched the whole surface of attachment with chromic acid fused on the end of a concealed probe. In ten days the wound had healed and up to date now more than three years, there has been no signs of recurrence.

*Case Second*—C. K., aged nine, small of her age but well nourished. There was no family history of any constitutional disease as far as I was able to find out. This little miss had been troubled with hoarseness ever since she was three years old and was gradually growing worse. During the past year there has been some difficulty in breathing so as to interfere greatly with her taking exercise. She was also losing flesh when she came under my observation. On laryngoscopic examination I found that she was suffering from a chronic laryngitis and pharyngitis, so that the throat was extremely irritable, causing great difficulty in getting a good view of the parts. In the larynx I found a rough, nodular growth extending from the anterior commissure half way back into chink of the glottis and a second growth starting from the left ventricular band and overlapping the left cord. Neither growth had any attachment to the cords. After considerable training, which consisted in passing a large rubber probe, so as to accustom her to the manipulation of instruments, I operated, using Schroeter's snare, caught the growth starting from the ventricular band and removed it entire without loss of blood and no pain. This growth was five lines long, three wide and two thick. It resembled greatly a small, white mulberry. The next day by the same means I removed the portion between the cords so that speech was restored, but I was greatly surprised to find that large mass still remained below the cords. This I found very difficult to reach owing to the small size of the larynx and the growth being attached to the anterior surface of the larynx and trachea. After repeated efforts I succeeded in removing all of this growth with a sharp curette, the tumor being quite friable. The tumors all being removed the whole surface of attachment was touched with a saturated solution of salicylic acid in water. Since that time I have removed several small growths from the same patient at intervals of two or three months.

Both these cases presented great difficulties in operation owing to the small size of the laryngies and the attachment of the growths. Then they were interesting as presenting two types of papillomata, one recurrent and the other non-recurrent. Another peculiarity was that in both cases the voice was affected before the beathing was interfered with.

